# Uptake, Accuracy, Safety, and Linkage into Care over Two Years of Promoting Annual Self-Testing for HIV in Blantyre, Malawi: A Community-Based Prospective Study

**DOI:** 10.1371/journal.pmed.1001873

**Published:** 2015-09-08

**Authors:** Augustine T. Choko, Peter MacPherson, Emily L. Webb, Barbara A. Willey, Helena Feasy, Rodrick Sambakunsi, Aaron Mdolo, Simon D. Makombe, Nicola Desmond, Richard Hayes, Hendramoorthy Maheswaran, Elizabeth L. Corbett

**Affiliations:** 1 Malawi–Liverpool–Wellcome Trust Clinical Research Programme, Blantyre, Malawi; 2 Department of Public Health and Policy, University of Liverpool, Liverpool, United Kingdom; 3 Department of Clinical Sciences, Liverpool School of Tropical Medicine, Liverpool, United Kingdom; 4 London School of Hygiene & Tropical Medicine, London, United Kingdom; 5 HIV Unit, Ministry of Health, Lilongwe, Malawi; 6 Division of Health Sciences, Warwick Medical School, Coventry, United Kingdom; Massachusetts General Hospital, Harvard Medical School, UNITED STATES

## Abstract

**Background:**

Home-based HIV testing and counselling (HTC) achieves high uptake, but is difficult and expensive to implement and sustain. We investigated a novel alternative based on HIV self-testing (HIVST). The aim was to evaluate the uptake of testing, accuracy, linkage into care, and health outcomes when highly convenient and flexible but supported access to HIVST kits was provided to a well-defined and closely monitored population.

**Methods and Findings:**

Following enumeration of 14 neighbourhoods in urban Blantyre, Malawi, trained resident volunteer-counsellors offered oral HIVST kits (OraQuick ADVANCE Rapid HIV-1/2 Antibody Test) to adult (≥16 y old) residents (*n =* 16,660) and reported community events, with all deaths investigated by verbal autopsy. Written and demonstrated instructions, pre- and post-test counselling, and facilitated HIV care assessment were provided, with a request to return kits and a self-completed questionnaire. Accuracy, residency, and a study-imposed requirement to limit HIVST to one test per year were monitored by home visits in a systematic quality assurance (QA) sample.

Overall, 14,004 (crude uptake 83.8%, revised to 76.5% to account for population turnover) residents self-tested during months 1–12, with adolescents (16–19 y) most likely to test. 10,614/14,004 (75.8%) participants shared results with volunteer-counsellors. Of 1,257 (11.8%) HIV-positive participants, 26.0% were already on antiretroviral therapy, and 524 (linkage 56.3%) newly accessed care with a median CD4 count of 250 cells/μl (interquartile range 159–426). HIVST uptake in months 13–24 was more rapid (70.9% uptake by 6 mo), with fewer (7.3%, 95% CI 6.8%–7.8%) positive participants. Being “forced to test”, usually by a main partner, was reported by 2.9% (95% CI 2.6%–3.2%) of 10,017 questionnaire respondents in months 1–12, but satisfaction with HIVST (94.4%) remained high. No HIVST-related partner violence or suicides were reported. HIVST and repeat HTC results agreed in 1,639/1,649 systematically selected (1 in 20) QA participants (99.4%), giving a sensitivity of 93.6% (95% CI 88.2%–97.0%) and a specificity of 99.9% (95% CI 99.6%–100%). Key limitations included use of aggregate data to report uptake of HIVST and being unable to adjust for population turnover.

**Conclusions:**

Community-based HIVST achieved high coverage in two successive years and was safe, accurate, and acceptable. Proactive HIVST strategies, supported and monitored by communities, could substantially complement existing approaches to providing early HIV diagnosis and periodic repeat testing to adolescents and adults in high-HIV settings.

## Abstract 2

In a prospective study, Augustine Choko and colleagues assess uptake, accuracy, and outcomes of implementation of community-wide HIV self-testing delivered and supported by resident community volunteers in 14 neighborhoods of Blantyre, Malawi.

## Editors' Summary

**Background:**

Every year, about 2.1 million people (70% of whom live in sub-Saharan Africa) are newly infected with HIV, the virus that causes AIDS, and 1.5 million people (again, mainly in sub-Saharan Africa) die as a result. HIV, which is usually transmitted through unprotected sex with an infected individual, gradually destroys CD4 lymphocytes and other immune system cells, leaving HIV-positive individuals susceptible to other serious infections and to unusual cancers. HIV is diagnosed by looking for antibodies to HIV in blood or saliva. After diagnosis, the progression of HIV infection is monitored by regularly counting the number of CD4 cells in the blood. Initiation of antiretroviral therapy—a combination of drugs that keeps HIV replication in check but that does not cure the infection—is recommended when an individual’s CD4 count falls below 500 cells/μl or when he or she develops an AIDS-defining condition.

**Why Was This Study Done?:**

HIV-positive individuals need to know their status so that they can take steps to avoid transmitting the virus to other people (for example, by always using a condom during sexual intercourse) and so that they can begin treatment. Treatment helps to keep HIV-positive individuals healthy but also reduces their chances of transmitting the virus to their sexual partners. Unfortunately, many HIV-positive individuals are unaware of their status. The situation is particularly bad in sub-Saharan Africa, where, despite major investments in facility-based and community-based HIV testing and counseling (HTC) programs, only a quarter of adults have had a recent HIV test, and only half of the people living with HIV know they are HIV positive. Barriers to facility-based HTC include concern about lack of confidentiality and fears of stigmatization. Home-based HTC avoids some of these barriers and can achieve high uptake of testing, but doubts have been expressed about the sustainability of this approach to testing. Here, the researchers evaluate an alternative to home-based HTC—HIV self-testing (HIVST)—by undertaking a community-based prospective study of HIVST in Blantyre, Malawi. HIVST involves individuals performing and interpreting their own HIV test and has the potential to be widely implemented with minimal involvement of trained healthcare workers.

**What Did the Researchers Do and Find?:**

Trained resident volunteer-counselors offered one oral HIVST kit (a kit that measures HIV in saliva) per year for a two-year period to 16,660 adult residents in 14 neighborhoods in urban Blantyre. All the participants received instructions on how to use the kits, pre- and post-counseling, and, for participants self-testing HIV positive, a referral card to attend an HIV care clinic. The residents also completed a questionnaire about their experience of HIVST. Three-quarters of the residents self-tested in the first and second year of the study. HIVST uptake was more rapid in the second year than in the first year and was high among men and adolescents, two hard-to-reach populations. Three-quarters of the residents who self-tested during the first year of the study shared their results with a volunteer-counselor. Of the 1,257 participants who discovered they were HIV positive during the first year of the study, more than half accessed HIV care. Importantly, 94.4% of the participants reported that they were happy with HIVST even though 2.9% reported being forced to take the test, usually by a main partner; no HIVST-related partner violence or suicides were reported by the study’s community surveillance system. Finally, HIVST and repeat HTC results agreed in 99.4% of participants selected as a quality assurance sample (one in 20 of the participants).

**What Do These Findings Mean?:**

These findings show that, in urban neighborhoods in Malawi, coverage with community-based HIVST was high (particularly among adolescents and men) in two successive years and that HIVST was safe, accurate, and acceptable. Importantly, HIVST using a delivery model based on trained volunteers led to acceptable linkage into HIV care services, and the approach had a very low incidence of major social harms such as partner violence. Uncertainty about estimates of uptake and linkage to care and other aspects of the study design may limit the accuracy of these results. Nevertheless, these findings suggest that scaling up HIVST could complement existing strategies for providing early HIV diagnosis and periodic repeat testing and could thus have a sustained impact on the coverage of HIV testing and care in Africa and on the control of the HIV/AIDS epidemic.

**Additional Information:**

This list of resources contains links that can be accessed when viewing the PDF on a device or via the online version of the article at http://dx.doi.org/10.1371/journal.pmed.1001873.

- Information is available from the US National Institute of Allergy and Infectious Diseases on all aspects of HIV infection and AIDS, including testing and diagnosis
- NAM/aidsmap provides basic information about HIV/AIDS, summaries of recent research findings on HIV care and treatment, and personal stories about living with HIV/AIDS
- Information is available from Avert, an international AIDS charity, on many aspects of HIV/AIDS, including HIV testing, HIV/AIDS treatment and care, and HIV prevention, and on HIV/AIDS in Malawi and in sub-Saharan Africa; Avert also provides personal stories about living with HIV/AIDS
- The World Health Organization provides information on all aspects of HIV/AIDS (in several languages), including its new consolidated guidelines on HIV testing
- The UNAIDS Fast-Track Strategy to End the AIDS Epidemic by 2030 provides up-to-date information about the AIDS epidemic and efforts to halt it; UNAIDS also provides detailed region-specific information and policy news.

## Introduction

Sub-Saharan Africa is still disproportionately affected by the HIV epidemic, accounting for 71% (24.7 million) of people living with HIV globally; in 2013, 71% of the 2.1 million global new infections, and 73% of the 1.5 million HIV-related deaths, occurred in the region [1]. Despite major investments in HIV testing, treatment, and prevention programmes, only one-quarter of adult Africans have had a recent HIV test, and half of people living with HIV in sub-Saharan Africa do not know they are HIV positive [1–3].

Barriers to HIV testing and counselling (HTC) and initiation of antiretroviral therapy (ART) include overly busy health facilities, concerns about lack of confidentiality and privacy, and high out-of-pocket costs [4–6]. Community-based HTC approaches, including home-based and mobile services, can overcome some of these problems, achieving high population uptake of HTC [7–10]. Compared to facility-based approaches, community-based HTC provides earlier HIV diagnosis and increases uptake of couples testing [4,5]. Nevertheless, evaluation of community-based HTC and HIV services has raised concerns about cost and sustainability [11,12], especially for delivering services to more rural settings [12,13]. For example, despite community-based HTC being national policy in Malawi and Zimbabwe, only 2% of Malawians and 4% of Zimbabweans in 2010 were reached by mobile or door-to-door services [3].

HIV self-testing (HIVST), defined as an individual performing and interpreting his/her own HIV test [14], has the potential to be implemented at a wide scale with a minimal requirement for trained health-workers. As such, HIVST could improve population coverage of regular HTC, recognised as being a critical component of all strategies to further intensify HIV prevention and care in countries with generalised HIV epidemics. We have previously demonstrated very high uptake and accuracy of HIVST in a small feasibility study [7]. However, critical, unanswered questions that need to be addressed before considering large-scale interventions based on HIVST include the following: what levels of HIVST uptake and accuracy can be achieved with population-wide implementation, and do safety concerns, including the potential for coercive testing, suicide, and gender-based violence, preclude implementation [15–17]?

We, therefore, investigated the uptake, accuracy, and outcomes of implementation of community-wide HIVST delivered by trained resident volunteer-counsellors in Blantyre, Malawi [18]. A delivery system based on service provision from the houses of volunteer-counsellors was designed. The aim was to evaluate uptake, accuracy, linkage into care, and health outcomes when highly convenient and flexible but supported access to HIVST kits was provided to a well-defined and closely monitored population. HIVST services were flexibly provided, with facilitated access to HIV care for those willing to share positive results. Participants could opt for support ranging from standard provider-conducted HTC to HIVST at home either in complete privacy or assisted by an attendant volunteer-counsellor.

## Methods

### Ethical Statement

Ethical approval was obtained from the College of Medicine Ethics Review Committee, University of Malawi; London School of Hygiene & Tropical Medicine; and Liverpool School of Tropical Medicine. All participants opting for HIVST provided written (or witnessed thumbprint) informed consent.

### Study Design

This study was a prospective study nested within a cluster-randomised trial (ISRCTN02004005) comparing health outcomes between 14 clusters randomised to HIVST and 14 clusters randomised to routine (facility-based) HTC [18]. The data reported here relate only to the 14 clusters where HIVST was provided. HIVST was provided for a 2-y period in any given cluster, starting between February and May 2012; active surveillance for harms continued for 4–6 mo after the 2-y HIVST period.

### Study Setting and Study Population

The study took place in three high-density informal residential settlements in urban Blantyre, as described elsewhere [10,18]. In brief, neighbourhood clusters were defined on the basis of existing community health worker catchment areas and enumerated between April and June 2011. In clusters randomised to the intervention arm, community-based HIVST was available for all adults (≥16 y). Services were provided by two resident volunteer-counsellors in each cluster of ~1,200 adults; the volunteer-counsellors were identified using participatory methods [19] and were paid a monthly stipend similar to that of Malawi Ministry of Health community health workers. Volunteer-counsellors received Malawi Ministry of Health HTC training and study-specific HIVST and protocol training. Targets within each cluster were to reach >80% of adult residents each year through promoting HIVST door to door and leafleting. Participants could opt to test at home, with or without the volunteer-counsellor present to provide help as needed.

### HIV Self-Testing Kit Provision

Participants (individuals or couples) received pre-test counselling, received instructions on performing HIVST, and were asked to demonstrate understanding using a cotton bud and vial of water in place of the kit itself. An anonymous self-completed questionnaire (SCQ) was provided with an opaque envelope for return of the used kit and SCQ, either to the volunteer-counsellor or into a locked “ballot” box kept at the volunteer-counsellor’s house (S1 Questionnaire). The test kit used was OraQuick ADVANCE Rapid HIV-1/2 Antibody Test (OraSure Technologies). User instructions were modified and included pictures. The ten-item SCQ included questions about the self-read HIVST result, satisfaction indicators, and the results of the individual’s most recent previous HIV test, if applicable. The question “If you were forced to test, who forced you?” was used to define coercion. Residents were asked to limit HIVST to one test in each 12-mo time period. Post-test counselling was recommended, but not required. All participants received a “self-referral card” allowing them to directly access one of two study clinics, but were encouraged to share results with their resident volunteer-counsellor for standard results-based post-test counselling and referral. A modified counselling protocol (including written information on all local HIV care options) was used for participants unwilling to share their results.

Within seven of the 14 study clusters, a second cluster-randomised controlled trial was conducted that investigated the effect of optional home-based initiation of HIV care (ART eligibility assessment and 2 wk of treatment including ART if indicated) on uptake of ART [10]. This intervention was extended to all 14 HIVST clusters from January 2013 onwards.

At health facilities, a study nurse provided confirmatory HIV testing (Determine HIV-1/2, Alere; and Uni-Gold Recombigen HIV, Trinity Biotech), CD4 count measurement (Cyflow SL-3 platform, Partec), tuberculosis screening (with isoniazid preventive therapy for those eligible [20]), WHO clinical staging, and cotrimoxazole. Participants who met national ART eligibility criteria (CD4 count < 350 cells/μl or WHO stage 3 or 4 or breastfeeding or pregnant) were registered for ART.

### Ascertainment of Outcomes

Volunteer-counsellors recorded each individual/couple with nature of support provided for the test, age, and sex of the individual(s), and whether they had tested before. Estimates of linkage into care were based on the number of participants who disclosed positive results to counsellors during the first 12 mo compared to the number of participants accessing study clinic confirmatory testing and HIV care over the same time period. Confirmation of participation in the study was based on presentation of the self-referral card.

### Recording Social Harms

In each cluster, four community members (key informants) provided weekly reports of all deaths and any known episodes of intimate partner violence. Study nurses conducted verbal autopsies for all reported deaths, including temporal relatedness to HIVST.

### Quality Assurance

A systematic sample of HIVST participants was selected for home visit by study nurses, aiming for minimum 5% coverage. Nurses selected from participants tested in the previous week using counsellors’ HIVST logs that recorded one participant or couple per row, with 20 rows per page. A random number between 1 and 20 was generated on a weekly basis and provided to nurses on the day of use. Nurses selected the corresponding row number (e.g., each row 11 participant if the number 11 had been supplied that week). If the selected number exceeded the number of participants on any given page, then the nurses continued counting out from row 1 of the same page until that week’s number was reached. Checks during the home visit included age, confirmation of residency, whether or not HIVST kits had been used, and self-read result, with offer of confirmatory testing (finger-prick blood parallel testing with Determine HIV-1/2 and Uni-Gold Recombigen HIV).

### Statistical Analysis and Sample Size

Stata version 13.0 (StataCorp) and R version 2.15.3 (R Foundation for Statistical Computing) were used for analyses. The sample size for the parent cluster-randomised trial was determined by the primary outcome (cluster-level tuberculosis case notification rates) and not by HIVST uptake or linkage. Of note, however, primary outcome assumptions were that population uptake of HIVST would be ≥70% per year [7,8], with ≥80% linkage into HIV care [21] and HIVST accuracy of ≥90% [7].

The proportion of residents accepting HIVST was estimated both overall and within sex, age, and neighbourhood strata, using population denominators from the study census (i.e., proportions were calculated using a fixed denominator that was determined before the start of the study, rather than as cumulative incidence, which would have required individual cohort follow-up for all residents) conducted in the year preceding the rollout of the intervention. Since crude uptake in some sex-age-neighbourhood subgroups exceeded the population denominators from the study census, the number of residents accepting HIVST within any single sex-age-neighbourhood subgroup was capped at the census denominator for that subgroup to provide an adjusted uptake.

The first estimate of linkage into care was calculated with the number of participants who presented at a study clinic with a volunteer-counsellor-provided self-referral card as the numerator and the number of participants who disclosed a positive HIV result to the volunteer-counsellor as the denominator. The second estimate was calculated after adjusting for a proportion assumed to be already aware of their positive HIV status and in care.

Participant characteristics in months 1–12 and months 13–24 were compared using design-based F-tests calculated by applying the second-order Rao and Scott correction [22,23] to the usual Pearson chi-squared test statistic for two-way tables to allow for the clustered sampling design. The accuracy of self-reported HIVST results in quality assurance (QA) participants was assessed using finger-prick rapid diagnostic test results to calculate sensitivity, specificity, and exact binomial 95% confidence intervals. Univariate and multivariate random effects logistic regression models accounting for clustering at the neighbourhood level were fitted in order to obtain odds ratios (ORs) and 95% CIs for associations between prespecified exposures of interest (age, sex, previous testing, testing alone/with partner, self-read HIVST result) and reported coercion. A substantial proportion of SCQ participants had missing data for at least one of the exposures of interest. Comparison of characteristics of participants with and without complete data showed no significant differences, and, therefore, findings from complete case analysis are presented [24]. Sensitivity analysis was undertaken using multiple imputation methods to handle missing data.

## Results

### Uptake of HIVST

**Fig 1:**
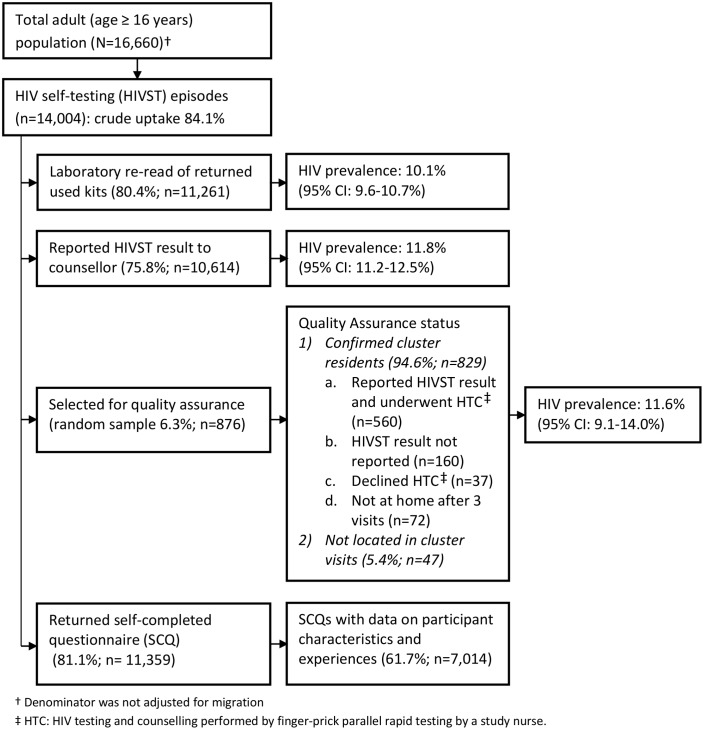
Flow of study participants in months 1–12 of HIV self-testing.

**Fig 2:**
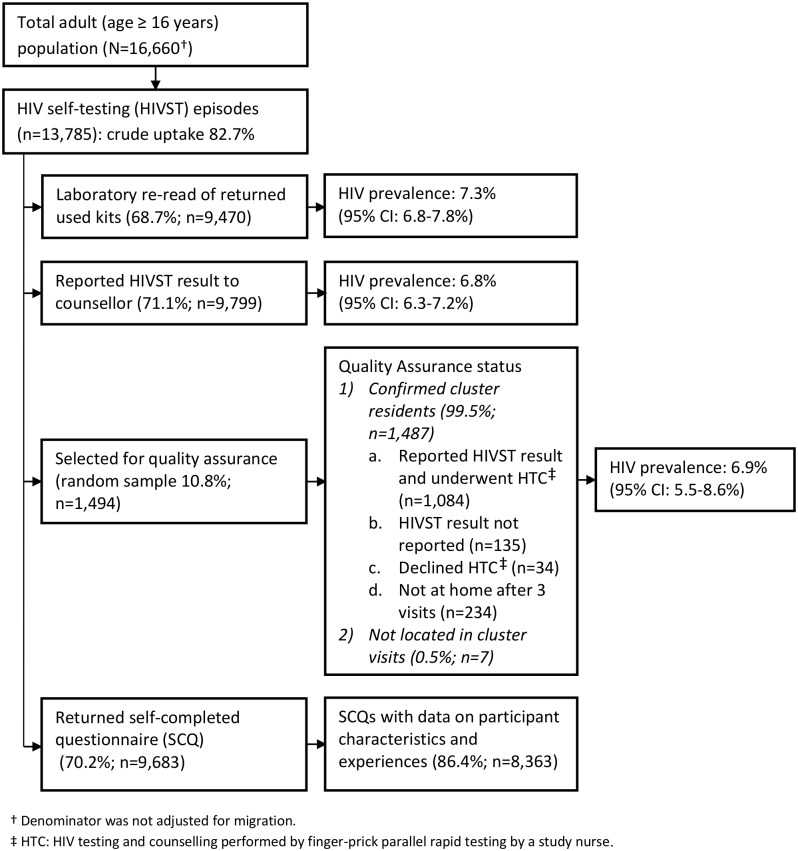
Flow of study participants in months 13–24 of HIV self-testing.

In 2011, 16,660 adults (16 y or older) were enumerated in the 14 HIVST clusters. During months 1–12 and months 13–24, a total of 14,004 (84.1%) and 13,785 (82.7%) participants accessed the HIVST service, respectively (Figs 1 and 2). Compared to months 1–12, the second year saw higher proportions of men (46.1% versus 43.8%; *p* = 0.057), adolescents (24.7% versus 22.2%; *p* < 0.001), participants with a sexual partner (59.3% versus 37.5%; *p* < 0.001), and participants who had tested for HIV ever (82.2% versus 64.9%, and for testing within the last 12 mo, 61.2% versus 27.3%; *p* < 0.001 for both) (Table 1).

**Table 1:** Characteristics of HIV self-testing participants in the first and second years of HIV self-testing availability.

Characteristic	Uptake of HIVST				*p*-Value^1^
	Month 1–12(*n* = 14,004)		Month 13–24(*n* = 13,785)		
	*n*	Percent	*n*	Percent	
**Sex**					
Male	6,124	43.8	6,339	46.1	0.057
Female	7,868	56.2	7,415	53.9	
**Age group**					
<20 y	3,107	22.2	3,399	24.7	<0.001
20–29 y	6,375	45.6	6,381	46.3	
30–39 y	2,995	21.4	2,806	20.4	
40–49 y	897	6.4	730	5.3	
≥50 y	597	4.3	431	3.1	
**Able to read and write?**					
No	742	5.3	366	2.7	0.002
Yes	13,124	94.7	13,090	97.3	
**Ever previously tested for HIV?**					
No	4,893	35.1	2,427	17.8	<0.001
Yes	9,040	64.9	11,205	82.2	
**Tested for HIV in last 12 mo?**					
No	10,034	72.7	5,217	38.8	<0.001
Yes	3,771	27.3	8,227	61.2	
**Ever self-tested for HIV before?**					
No	13,509	97.9	7,508	55.9	<0.001
Yes	290	2.1	5,931	44.1	
**Tuberculosis symptoms?** ^2^					
No	13,301	96.8	13,357	98.7	<0.001
Yes	434	3.2	178	1.3	
**Who initiated testing?** ^3^					
Client	5,405	38.8	3,163	23.1	0.075
Counsellor	8,543	61.2	10,506	76.9	
**Have a sexual partner?**					
No	6,826	62.5	3,520	40.7	<0.001
Yes	4,098	37.5	5,128	59.3	

^1^ *p*-Value from design-based F-test allowing for clustering by neighbourhood of residence.

^2^Having any of the following: cough of any duration, fever, night sweats, or weight loss.

^3^The client was considered to have initiated testing if the client visited the community counsellor explicitly to request an HIVST kit; the counsellor was considered to have initiated testing if the community counsellor visited the client at the client’s home either by prior arrangement or during door to door rounds.

The estimated uptake of HIVST, based on study census denominators, was 84.1% and 82.7% in months 1–12 and months 13–24, respectively. Crude uptake in some age-sex-neighbourhood subgroups (notably among adolescent women [aged 16–19 y]) exceeded population denominators from the census conducted in the year preceding the study (Table 2). Capping uptake in any single age-sex-neighbourhood subgroup at 100% led to revised uptake estimates of 76.5% and 74.4% in months 1–12 and months 13–24, respectively. With both approaches, there was significantly higher uptake each year amongst women than men, and for progressively younger age groups (*p* < 0.001 for both).

**Table 2:** Age-sex distribution of study population and study participants with and without adjustment by study census maximum denominators in age-sex-neighbourhood subgroups.

Characteristic	Study Census	Crude Uptake^1^			Revised Uptake^2^		
		HIVST Uptake	Percent	*p*-Value	HIVST Uptake	Percent	*p*-Value
**Months 1–12 of HIVST**							
***Total***	16,660	14,004	84.1	—	12,751	76.5	—
***Men***							
16–19 y	1,196	1,223	102.3	<0.001	1,068	89.3	<0.001
20–29 y	3,326	2,686	80.8		2,646	79.6	
30–39 y	2,462	1,491	60.6		1,477	60.0	
40–49 y	926	412	44.5		412	44.5	
≥50 y	733	299	40.8		299	40.8	
***Women***							
16–19 y	1,306	1,884	144.3	<0.001	1,306	100.0	<0.001
20–29 y	3,487	3,682	105.6		3,313	95.0	
30–39 y	1,872	1,502	80.2		1,458	77.9	
40–49 y	627	484	77.2		461	73.5	
≥50 y	510	297	58.2		297	58.2	
***Either sex or age missing***	215	44	20.5		14	6.5	
**Months 13–24 of HIVST**							
***Total***	16,660	13,785	82.7	—	12,396	74.4	—
***Men***							
16–19 y	1,196	1,382	115.6	<0.001	1,104	92.3	<0.001
20–29 y	3,326	2,892	87.0		2,828	85.0	
30–39 y	2,462	1,448	58.8		1,412	57.4	
40–49 y	926	364	39.3		348	37.6	
≥50 y	733	235	32.1		232	31.7	
***Women***							
16–19 y	1,306	2,010	153.9	<0.001	1,301	99.6	<0.001
20–29 y	3,487	3,475	99.7		3,270	93.8	
30–39 y	1,872	1,354	72.3		1,331	71.1	
40–49 y	627	363	57.9		353	56.3	
≥50 y	510	195	38.2		190	37.3	
***Either sex or age missing***	215	67	31.2		27	12.6	

^1^For each sex-age group, the number of people in that group who tested through HIVST (years: 2012–2014) is the numerator, and the total number of people in that sex-age group at the time of census (2011) is the denominator. Uptake estimate may exceed 100% due to population turnover.

^2^For each sex-age group, the numerator is the number of people in that group who tested through HIVST (years: 2012–2014) but now capped at the census denominator for that sex-age group for those age-sex groups where the number of testers exceeded the number of people in that group in the census. The denominator is the total number of people in that sex-age group at the time of census (2011).

^3^Chi-squared test for HIVST yes/no.

**Fig 3:**
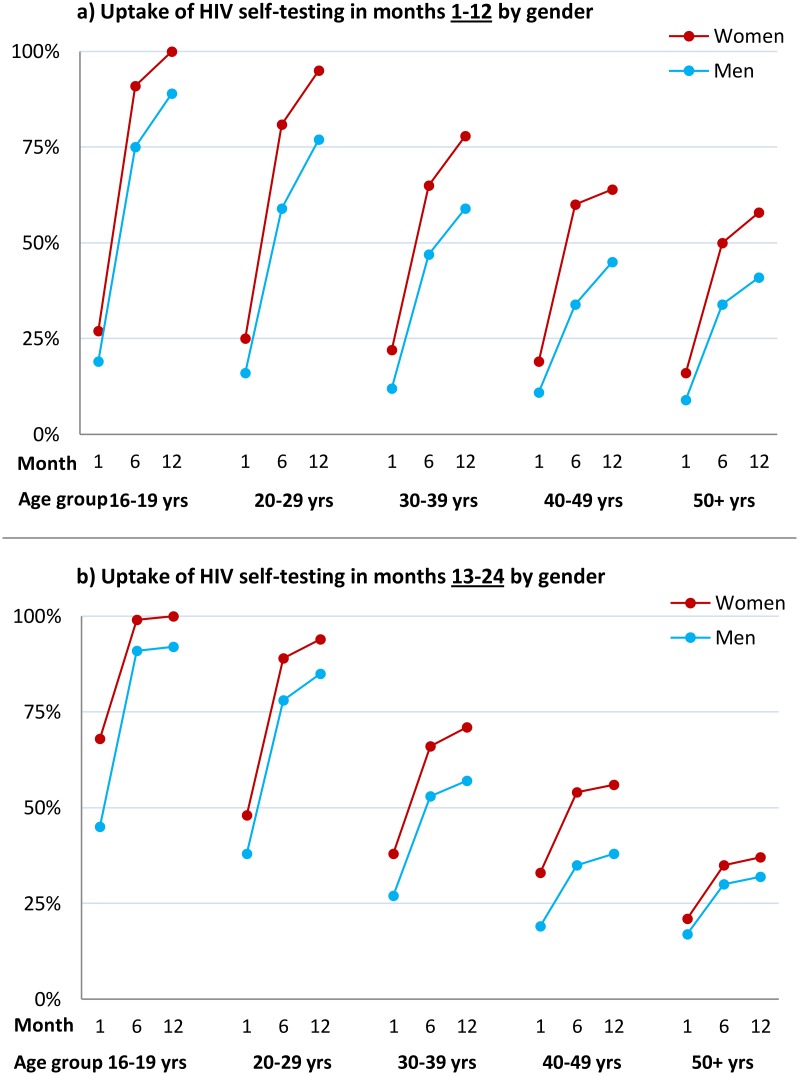
Cumulative uptake of HIV self-testing by sex, age group, and time point. (A) Cumulative uptake of HIVST during the first 12 mo of availability among all HIVST cluster residents by age and time point among men and women. HIVST uptake increased with time, rising to close to 100% by 12 mo in adolescents (age group 16–19 y); uptake for men was lower than for women at every time point. (B) Cumulative uptake of HIVST during months 13–24 of HIVST availability among all cluster residents by age and time point. Uptake defined as an individual having collected an HIVST kit from a community counsellor. Since crude uptake of HIVST exceeded 100% in some age-sex-neighbourhood subgroups, likely explained by migration, revised estimates were calculated where uptake in any single age-sex-neighbourhood subgroup was censored at 100%; study census data were used for denominators.

The time course of HIVST uptake within each annual period for which HIVST was restricted to a single test per person (Methods and QA results) is shown by time point, sex, and age group in Fig 3. In comparison to months 1–12, uptake during the second year of availability was more rapid, with a higher proportion accessing services soon after they became available (Fig 3), notably so for adolescents (aged 16–19 y).

### HIV Prevalence in HIVST Participants and Linkage into Care

**Fig 4:**
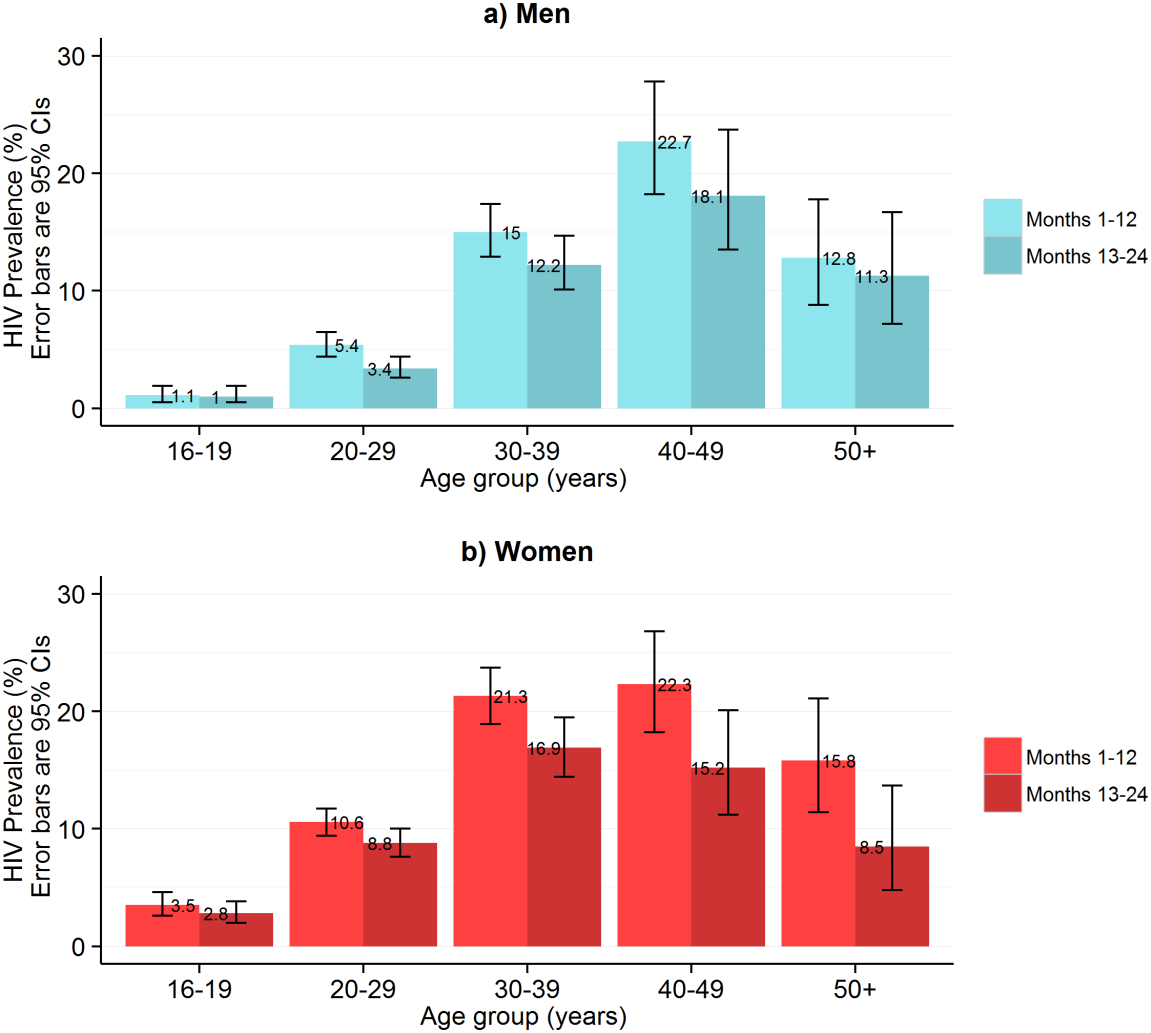
HIV prevalence in self-testing participants who returned used test kits by sex and age group and time of HIV self-testing availability. This figure shows HIV prevalence in HIVST participants for men (A) and women (B), stratified by time of HIVST availability. Bars show HIV prevalence (percent); error bars show 95% confidence intervals. Estimates are based on denominators determined through enumeration. Numerators were based on a reread of used and returned HIVST kits by a laboratory technician within 2 wk of use. Individuals were asked to test only once within each 12-mo time period, and retesting in people already aware of their positive HIV status was discouraged.

In the first year of HIVST, HIV prevalence in participants sharing results with volunteer-counsellors was 11.8% (95% CI 11.2%–12.5%), similar to the estimate from the rereading of returned kits (10.1%, 95% CI 9.6%–10.7%) (Fig 1). These estimates, however, were substantially higher than the respective figures from months 13–24, which were 6.8% (95% CI 6.3%–7.2%) and 7.3% (95% CI 6.8%–7.8%). HIV prevalence among self-testing participants (shown separately for men and women in Fig 4) was highest in the age group 40–49 y, with a pooled prevalence of 22.5% (95% CI 19.4%–25.8%) in months 1–12; the pooled rate in participants aged 16–19 y (2.5%, 95% CI 1.9%–3.2%) was much lower.

**Fig 5:**
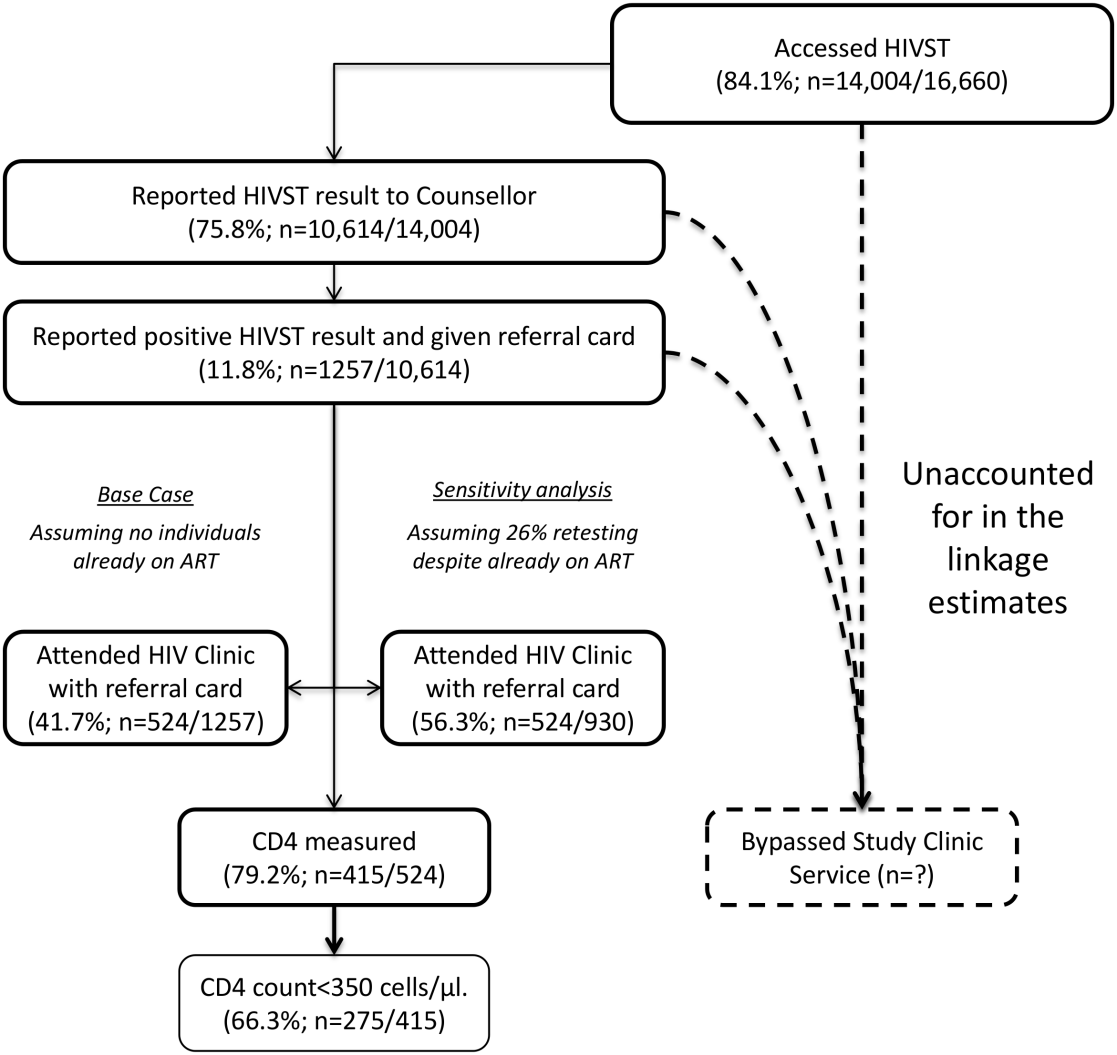
Linkage into HIV care after HIV self-testing (months 1–12).

In total, 75.8% (95% CI 75.1%–76.5%; 10,614/14,004) of participants who underwent HIVST in months 1–12 reported their result to a volunteer-counsellor, with 1,257 (11.8%, 95% CI 11.2%–12.5%) reporting a positive result. During this same time period, 524 participants presented for HIV care, with all presenting cards identifying them as having been directly referred in by a volunteer-counsellor (Fig 5). Thus, our first estimate of linkage is 41.7% (524 of 1,257 self-testing positive). However, in a subset of 3,016 participants in months 1–12, 2,380 (78.9%; 95% CI 77.4%–80.4%) responded to a question about ART. Of these, 219 (9.2%, 95% CI 8.1%–10.4%) were HIV positive, and 57 (26.0%, 95% CI 20.3%–32.4%) of these individuals stated that they were already on ART, consequently increasing our estimate of linkage to 56.3% (524/930). The median CD4 count from 415 participants (72.9% of those attending care) was 250 cells/μl (interquartile range [IQR] 159–426), with 66.3% (275/415) of CD4 counts being below 350 cells/μl.

### Accuracy

A total of 2,361 (8.5%) of 27,789 HIVST participants were included in QA tracing (shown for separate years in Figs 1 and 2). Only 54 (2.3%) were found not to be cluster residents, while 1,649 (69.8%) agreed to confirmatory HIV testing. Results were positive in 141 (8.6%, 95% CI 7.2%–10.0%). Compared to stated HIVST results, there were 9/1,508 (0.6%) false negatives (including four participants already on ART) and 1/133 false positives, giving agreement of 1,639/1,649 (99.4%, 95% CI 98.9%–99.7%), sensitivity of 93.6% (95% CI 88.2%–97.0%), and specificity of 99.9% (95% CI 99.6%–100%) (Table 3).

**Table 3:** Summary of quality assurance process and accuracy results.

Self-Reported HIV Self-Test Result	Index Test*		
	Positive	Negative	Total
Positive	132	1	133
Negative	9**	1,507	1,516
Total	141	1,508	1,649

Concordance: 99.4% (95% CI 98.9%–99.7%); sensitivity: 93.6% (95% CI 88.2%–97.0%); specificity: 99.9% (95% CI 99.6%–100.0%).

*Parallel testing with two rapid finger-prick blood tests by a trained nurse.

**Includes four participants later found to be already on ART.

### Acceptability of Self-Testing and Social Harms, Including Reported Coercive Testing

During months 1–12, 81.1% (95% CI 80.5%–81.8%; 11,359/14,004) participants returned a SCQ to the counsellor, with 7,014 (61.7%) completing all key fields including self-read HIVST result, coercion, and acceptability indicators (S1 Questionnaire). There was acceptable internal consistency (Cronbach’s alpha = 0.64) for the four variables relating to acceptability: overall satisfaction with HIVST, whether or not they would recommend HIVST to friends and family, how hard it was to self-test, and whether or not they trusted the results of an oral test [25].

Acceptability indicators were high in all age group and sex strata, with 94.6% (1,446/1,635) reporting that they were “highly satisfied” with the HIVST process and 97.1% (6,683/6,883) reporting they would “definitely recommend HIVST to their friends and family”. These indicators did not vary significantly by self-reported HIV status, with those testing positive having OR 0.60 (95% CI 0.34–1.05) and OR 0.92 (95% CI 0.56–1.50) relative to HIV-negative participants for being “very satisfied” with the HIVST process and for “definitely” recommending HIVST to friends and family, respectively.

In total, 288/10,017 participants (2.9%, 95% CI 2.6%–3.2%) reported having been coerced into participating in HIVST. Notably, however, satisfaction indicators in the group reporting coercion were high, with 94.4% (252/267) stating that they would recommend HIVST to friends and family, and 92.2% (130/141) reporting that they were highly satisfied with HIVST. In the univariate analysis, men and participants who self-tested with their partner were significantly more likely to report having been coerced into HIVST (Table 4). In multivariate analysis, male sex (adjusted OR [aOR] 1.83, 95% CI 1.38–2.43) and having tested with a partner (aOR 3.86, 95% CI 2.82–5.29) remained significantly associated with reported coercion. There was no significant difference in reporting of coercion by reported HIVST result to volunteer-counsellors (aOR 1.00, 95% CI 0.59–1.71). The findings were comparable when multiple imputation methods were used to handle missing data (S1 Table).

**Table 4:** Factors associated with reported coercion during months 1–12 of HIV self-testing (*n* = 7,014).

Characteristic	Number Coerced into HIVST/Total	Percent	OR^1^	95% CI^1^	aOR^1^	95% CI^1^
**Women**	91/4,138	2.2	1		1	
**Men**	112/2,868	3.9	1.81	1.36–2.39	1.83	1.38–2.43
**Age group**						
16–19 y	44/1,470	3.0	1		1	
20–29 y	102/3,276	3.1	1.04	0.73–1.49	1.05	0.73–1.50
30–39 y	47/1,499	3.1	1.05	0.69–1.59	1.01	0.66–1.53
40–49 y	6/446	1.4	0.44	0.19–1.04	0.44	0.18–1.03
≥50 y	4/315	1.3	0.42	0.15–1.17	0.39	0.14–1.10
**Ever tested before**	159/5,361	3.0	1		1	
**Never tested before**	44/1,645	2.7	0.90	0.64–1.26	0.86	0.60–1.23
**Self-tested alone**	136/6,157	2.2	1		1	
**Self-tested with partner**	67/849	7.9	3.8	2.80–5.13	3.86	2.82–5.29
**Self-test self-read result**						
Negative	182/6,299	2.9	1.00		1	
Positive	16/649	2.5	0.85	0.51–1.43	1.00	0.59–1.71
Don’t know	5/58	8.6	3.2	1.25–8.03	3.17	1.22–8.22
**Highly satisfied with HIVST**						
Yes	84/1,581	5.3	1.00		ND	ND
No	5/54	9.3	1.82	0.71–4.68	ND	ND
**Would recommend HIVST to friends and family**						
Yes	188/6,763	2.8	1.00		ND	ND
No	12/120	10.0	3.89	2.10–7.18	ND	ND

^1^ORs for age and sex were adjusted for each other only; ORs for all other variables were adjusted for age, sex, and each other.

ND, not done.

A total of 132 adult deaths were reported through the community liaison system during the first 12 mo of follow-up, including one suicide in an individual who had not self-tested and four murders, none of which had any known or close temporal relationship to self-testing. No intimate partner violence episodes were reported through the community liaison system.

## Discussion

The main finding of this study was the high population uptake of HIVST and retesting during 2 y of highly decentralised service provision in an urban community in Malawi. HIVST was safe and accurate, with uptake highest among adolescents, and with acceptable linkage into HIV care services using a delivery model based on trained volunteers. No suicides or other serious unintended consequences related to HIVST were detected by an active community surveillance system, including systematic death reporting and verbal autopsies. Feeling coerced into self-testing (usually by a main partner) was common (2.9% respondents), but was nonetheless associated with a high satisfaction rating for HIVST for all but a small minority of respondents. This model of HIVST is potentially scalable to other low-income settings where annual repeat HIV testing is recommended.

HIV testing needs in Africa have changed dramatically in the last decade due to the massive scale-up of ART services and an increasing focus on early diagnosis and treatment of HIV for prevention [26,27], as well as other biomedical HIV prevention strategies [28,29]. Population surveys and qualitative studies report high readiness to test, but there exist substantial barriers to accessing free clinic-based HIV testing services [30–33].

The high acceptability and ease of distribution of oral test kits makes HIVST of special interest in high-HIV settings, where the aim is to achieve affordable universal coverage and regular repeat testing [34]. Here we report considerable complementarity of this model of HIVST with existing strategies. Although our urban population was already served by free facility-based services, 35% of participants in the first 12 mo had never previously tested, and uptake was high in two important hard-to-reach groups: men and adolescents. Our estimates of adolescent population uptake (~100% for women aged 16–19 y and ~90% for men aged 16–19 y) are in stark contrast with reported adolescent HTC uptake in African DHS surveys [3]. Ideally, HIVST services would capitalise on high acceptability among key populations, facilitating linkage into HIV prevention programmes, such as pre-exposure prophylaxis and voluntary medical male circumcision, as well as ensuring prompt linkage into HIV care [14]. The per-episode costs of providing HIVST compared to the costs of facility-based testing will be reported fully elsewhere.

Our data from the second year of HIVST availability (participants were asked to test only once in each year) show high readiness to retest, as well as reduced numbers of first-time testers and new positive HIV diagnoses, which is consistent with the high coverage reported from the first year. Importantly, population uptake in the second year was faster, suggesting that under programmatic conditions, experienced volunteer-counsellors could cover larger populations as soon as communities have been familiarised with HIVST concepts.

Optimum systems for linking clients into HIV care/prevention programmes are not well established in Africa [35–38] but are critical to the public health impact and cost-effectiveness of HTC [39]. Here we estimate a timely linkage into confirmatory testing and HIV care following HIVST of 56%, which compares favourably with many other approaches [40] and is well within the expected range for African HTC services [35,36]. This linkage estimate, however, reflects that, in addition to HIVST, participants were asked to attend post-test counselling and were advised to share their HIVST results. Facilitated HIV care assessment and initiation was provided following a successful trial in the first 6 mo of this study [10]. Despite reluctance to be *tested* by a volunteer-counsellor who is a neighbour, willingness to take kits and to *share results* was high. Although at first seemingly paradoxical, other studies have also reported that learning one’s HIV status demands a moment of complete privacy, but that being able to turn to someone familiar can then make the next steps of accessing HIV care less daunting [41].

Some of the benefits of community-based HTC are reaching HIV-positive individuals earlier [42], improving survival [43], and reducing costs [44] and onward transmission. A recent meta-analysis has found that when CD4 measurement was offered in tandem with home-based HIV testing, approximately 60% of those who tested HIV positive had CD4 counts greater than 350 cells/μl [9]. Here we report a CD4 count profile below this ideal (median 250 cells/μl, IQR 159–426) for HIVST participants who subsequently attended care, but still considerably higher than that of HIV care attendees diagnosed from our study clusters following standard non-study HTC (median 154 cells/μl, IQR 116–249) [10].

Concerns about the potential impact of user error on diagnostic accuracy from HIVST [45,46] have been widely discussed [14]. Here we report an HIVST accuracy (93.6% sensitivity, 99.9% specificity) very similar to that of unobserved HIVST using the OraQuick ADVANCE Rapid HIV-1/2 Antibody Test in American participants [47]. We have previously reported 97.9% sensitivity and 100% specificity for a small observed/controlled-setting study in Blantyre [7]. In the HIVST model evaluated, users were given a short simple demonstration by trained lay volunteers, and this may have been a key factor in maintaining high accuracy in this relatively low literacy setting. Both accuracy and uptake of services post-testing will need revaluation if different test kits or less supportive models are considered, for example, over-the-counter or vending machine sales.

Also of note, a much higher than anticipated proportion (26%) of our HIV-positive HIVST participants were on ART already, as were two of our four participants found to have false-negative results. ART is known to reduce sensitivity especially for oral fluid-based rapid diagnostic tests [48]. In Malawi, faith healing, whereby HIV is considered curable through prayer, is widely preached and may prompt ART patients to reconsider their status and need for ART if they get a negative test result via HIVST [49]. Based on our experience, we would recommend careful messaging about retesting while on ART in HIVST package inserts and education campaigns.

Coercion was reported by 3% of our SCQ respondents and was the major social harm, with no suicides or intimate partner violence attributed to HIVST despite active surveillance. Comparable data suggest that feeling coerced affects other modalities of HTC, with an estimated 7% of HTC episodes in Africa occurring without consent [50]. Both pregnant women and their male partners commonly report feeling coerced into testing by health professionals [51]. Among our participants, men and those who tested with their partners were more likely to report coercion. HIVST programmes need to anticipate and guard against coercive and mandatory testing, and to ensure that information about rights is disseminated and that systems for reporting social harms are in place.

Study limitations include uncertainty around our linkage and uptake estimates, and use of aggregate-level data reporting rather than individual cohort follow-up. Population turnover, typically high in urban slums, was not factored into our population denominators, and may in part explain why our crude uptake estimates for adolescent women were >100%. Importantly, our QA programme results ruled out a major contribution to our findings from HIVST offered to non-eligible individuals (non-residents and individuals taking multiple tests). Estimates of linkage into care always have a wide uncertainty (Fig 5), but as disclosure of positive HIVST results was voluntary, even our precise denominators are unknown. Furthermore, we under-appreciated the extent of retesting while already on ART, adding to the uncertainty around numbers of newly identified HIV-positive participants. However, these sources of imprecision are unlikely to have affected our overall messages.

In summary, community-level HIVST service provision along with supportive post-test services resulted in high and rapid uptake of accurate HIVST, with very low incidence of major social harms, and acceptable linkage into HIV care. The continued high uptake in the second year suggests that scaling up HIVST could have a sustained impact on the coverage of HIV testing and care in Africa, especially for men and adolescents.

## Supporting Information

S1 Checklist STROBE checklist. (DOCX)

S1 Questionnaire Self-completed questionnaire. (PDF)

S1 Table Comparison between complete case analysis (*n* = 7,014) presented in Table 4 and analysis based on imputed data (*n =* 11,359). (DOCX)

## Abbreviations

aOR: adjusted odds ratio
ART: antiretroviral therapy
HIVST: HIV self-testing
HTC: HIV testing and counselling
IQR: interquartile range
OR: odds ratio
QA: quality assurance
SCQ: self-completed questionnaire
