# Supplementary material for: Uptake, Accuracy, Safety, and Linkage into Care over Two Years of Promoting Annual Self-Testing for HIV in Blantyre, Malawi: A Community-Based Prospective Study
Source: PLoS Med. 2015 Sep 8;12(9):e1001873. doi: 10.1371/journal.pmed.1001873 (PMC4562710; doi:10.1371/journal.pmed.1001873)
Supplement: S1 Questionnaire — (PDF) [file pmed.1001873.s002.pdf]

0774146155

## CP09: SELF COMPLETED EXIT INTERVIEW FORM

Gawo la wotenga mbali (Participant to fill this section)

**If you tested together as a couple, you still need to complete this form individually**

| | | |
|---|---|---|
| X01 AGE | **Kodi muli ndi zaka zingati?** My age is | ☐☐ Years |
| X02 SEX | **Ndine wa** I am | ☐ **Male (Mwamuna)** ☐ **Female (mkazi)** |
| X03 PID | **Self referral ID** | PLACE SELF REFERRAL BARCODE HERE |

| | | | | | |
|---|---|---|---|---|---|
| **EXAMPLE:** | **Wonetserani kwa otenga nawo mbali posankha yankho yolondola** Demostrate to the participant by ticking the appropriate box | **Nyengo ku Blantyre ili:** The weather in Blantyre is: | ☐ **Bwino** good | ☐ **Bwinobe** somewhat good | ☐ **Siyilibwino konse** Siyilibwino konse |
| X04 HIVTST | **Kodi munayamba mwayezetsapo kachilombo ka HIV?** Have you had an HIV test before? | | ☐ **Yes** | | ☐ **No** |
| X05 TST | **Ndinayezetsa** I last tested | | ☐ **On my own (Ndekha)** ☐ **with my partner (Ndi mkazi/mwamuna wanga)** | | |
| X06 RES | **Ngati munayezetsa,** zotsatira zake zinali:My last test result was | | ☐ **Positive** + | ☐ **Don't know** ? | ☐ **Negative** — |
| X07 ART | **Muli pamankhwala a ARV?** If HIV positive, are you on ART? | | ☐ **Yes** | | ☐ **No** |

<u>Chidziwitso :</u> Zotsatira zanu za zoyezetsa za HIV sizikhala ndi dzina lanu komanso palibe munthu amene azaziwe zotsatirazo olo aphungu amene pokhapokha mutawauza zotsatirazo inu nokha.

<u>Important :</u> Your HIV result will not be linked to your name and no one including the counsellor will know your result unless you disclose it yourself.

| | | | | |
|---|---|---|---|---|
| X08 RESCOR | **Kodi mukukhulupilira kuti zotsatira zanu poziyeza nokha zinali zoona?** Do you trust your self test result were correct ? | ☐ **Ndizoona** definately correct | ☐ **Mwina ndizoona** probably correct | ☐ **Sizoona** Not correct |
| X09 RES | **Zotsatira zanga ndi zoti:** My self-test result is: | ☐ **Ndilinako** positive + | ☐ **Sindikudziwa** not sure/invalid ? | ☐ **Ndilibe** negative — |
| X10 TSTHRD | **Kunali kovuta bwanji kuti mudziyeze molondola?** How hard was it for you to do the self-test correctly? | ☐ **Sikunali kovuta** Not at all hard to do tha test | ☐ **Kunali kovutilapo** somewhat hard to do the test | ☐ **Kunali kovuta kwambiri.** very hard to do the test |
| X11 SATIS | **Kodi ndinu wokhutitsidwa motani ndi ndondomeko yonse yoziyeza wekha?** Overall, how satisfied were you with the self-testing process | ☐ **Wokhutitsidwa** Very satisfied | ☐ **Wokhutitsidwa pang'ono** Somewhat satisfied | ☐ **Wosakhutitsidwa** Not satisfied |
| X12 RCMD | **Kodi mungawalimbikitse anzanu ndi ku banja kwanu kugwiritsa ntchito chipangizo choyezera cha mkamwachi?** Would you recommend this HIV test kit for self-testing to friends and family? | ☐ **Kwambiri** Definitely yes | ☐ **Kokayikitsa** Not sure | ☐ **Ayi** Definately no |

X13 OSPEC **Ngati munakakamizidwa kuyezetsa, ndi ndani anakukakamizani?** If forced to test, who forced you?

- ☐ **Okondedwa/mamuna wanga/mkazi wanga** (Husband/wife or spouce or partner)
- ☐ **Kholo/wondiyang'anira** (Parent or Guardian)
- ☐ **Achibale ena** (Other relative)
- ☐ **Mzanga/kapena ogwira naye ntchito** (Friend or colleague)
- ☐ **Sindinachite kukakamizidwa** (I was not forced)

X14 INFL **Ndikutiko mukufuna kuzakhale malo oyezetsera mtsogolo muno?** Which would you want most to be your next test?

- ☐ **Malo oyezetsera magazi** (VCT centre/hospital/clinic)
- ☐ **Kuyezetsa mothandizidwa ndi a phungu ku kunyumba** (Testing by a counsellor at home)
- ☐ **Kuyezetsa pamaso pa a phungu** (Self-testing in the presence of a counsellor)
- ☐ **Kuziyeza ndekha kunyumba**(Self-testing at home in private)

**PLEASE TURN OVER**

## CP09: SELF COMPLETED EXIT INTERVIEW FORM

Gawo la wotenga mbali (Participant to fill this section) **continues.......**

X15 IMPRV **Ndichiyani chomwe mukuona ngati chingapangitse kuti ndondomeko yoziyeza wekha ikhale yopambana?**
What would you recommend to improve the experience of self-testing?

---

The Community counsellor to fill this section if test kit seen
*(the counsellor will not read your result without your permission)*

X16 KITSN — Kit seen
- ☐ **Yes**
- ☐ **No**

X17 RES — Result
- ☐ **Negative**
- ☐ **Faint +ve**
- ☐ **Clear +ve**

X18 TSTREP — Test repeated
- ☐ **Yes**
- ☐ **No**

X19 REPRES — If repeated, test result
- ☐ **Negative**
- ☐ **Faint +ve**
- ☐ **Clear +ve**
- ☐ **Not seen**

For Lab Use

X20 TID **Technician's ID** ☐☐

X21 DTEC **Date form collected** ☐☐ ☐☐☐ 2 0 1 ☐
d d m m m y y y y

X22 RES **Lab**
- ☐ **R (clear)**
- ☐ **R (faint)**
- ☐ **Very faint** — **if very faint specify the colour** ☐☐☐☐☐☐☐☐☐☐
- ☐ **NR**
- ☐ **I**

X23 **KI** **Kit ID** ☐☐☐☐☐☐☐
