# Supplementary material for: Uptake, Accuracy, Safety, and Linkage into Care over Two Years of Promoting Annual Self-Testing for HIV in Blantyre, Malawi: A Community-Based Prospective Study
Source: PLoS Med. 2015 Sep 8;12(9):e1001873. doi: 10.1371/journal.pmed.1001873 (PMC4562710; doi:10.1371/journal.pmed.1001873)
Supplement: S1 Table — (DOCX) [file pmed.1001873.s003.docx]

**Table 5: Comparison between complete case analysis (N =7014) presented in Table 4 and analysis based on imputed data (N = 11,359)**

**Factors associated with reported coercion during months 1-12 of HIV self-testing**

|  | **Complete case** | | **Imputation** | | |  | | |  |  |
| --- | --- | --- | --- | --- | --- | --- | --- | --- | --- | --- |
| **Variable** | **OR** | **CI** | **OR** | **95% CI** | | | |  |  |  |
| Women | 1 |  | 1 |  | | | |  |  |  |
| Men | 1.83 | 1.38-2.43 | 1.77 | 1.40-2.23 | | | |  |  |  |
|  |  |  |  |  | | | |  |  |  |
| Age group |  |  |  |  | | | |  |  |  |
| 16-19 | 1 |  | 1 |  | | | |  |  |  |
| 20-29 | 1.05 | 0.73-1.50 | 1.26 | 0.93-1.73 | | | |  |  |  |
| 30-39 | 1.01 | 0.66-1.53 | 1.17 | 0.79-1.73 | | | |  |  |  |
| 40-49 | 0.44 | 0.18-1.03 | 0.75 | 0.41-1.40 | | | |  |  |  |
| 50+ | 0.39 | 0.14-1.10 | 0.54 | 0.24-1.19 | | | |  |  |  |
|  |  |  |  |  | | | |  |  |  |
| Ever tested before | 1 |  | 1 |  | | | |  |  |  |
| Never tested before | 0.86 | 0.60-1.23 | 0.80 | 0.59-1.10 | | | |  |  |  |
|  |  |  |  |  | | | |  |  |  |
| Self-tested alone | 1 |  | 1 |  | | | |  |  |  |
| Self-tested with partner | 3.86 | 2.82-5.29 | 4.10 | 3.12-5.41 | | | |  |  |  |
|  |  |  |  |  | | | |  |  |  |
| Self-test result: |  |  |  |  | | | |  |  |  |
| Negative | 1 |  | 1 |  | | | |  |  |  |
| Positive | 1.00 | 0.59-1.71 | 1.14 | 0.74-1.75 | | | |  |  |  |
| Don't know | 3.17 | 1.22-8.22 | 3.01 | 1.19-7.64 | | | |  |  |  |
| OR: odds ratio; CI: confidence interval | | | | | | | | | | |
| * Imputation by Chained Equations method | | | | |  | |  | | |  |
| ORs for age and sex were adjusted for each other only; ORs for all other variables were adjusted for age and sex | | | | | | | | | | |
